# Development of billing post competency evaluation index system for nurses in China: a Delphi study

**DOI:** 10.1186/s12912-023-01301-0

**Published:** 2023-04-25

**Authors:** Jiao Liu, Huifang Qiu, Xiaohong Zhang, Cuiling Zhang, Fang He, Pan Yan

**Affiliations:** 1 General Surgery Department, Shanxi Bethune Hospital, Shanxi Academy of Medical Sciences, Tongji Shanxi Hospital, Third Hospital of Shanxi Medical University, Taiyuan, 030032 China; 2 General Surgery Department, Tongji Hospital, Tongji Medical College, Huazhong University of Science and Technology, Wuhan, 430030 China; 3 Nursing Department, Shanxi Bethune Hospital, Shanxi Academy of Medical Sciences , Tongji Shanxi Hospital, Third Hospital of Shanxi Medical University, 99 Longcheng street, Taiyuan, 030032 Shanxi province China; 4 Nursing Department, Tongji Hospital, Tongji Medical College, Huazhong University of Science and Technology, Wuhan, 430030 China; 5 Department of Digestive Oncology, Shanxi Bethune Hospital, Shanxi Academy of Medical Sciences, Tongji Shanxi Hospital, Third Hospital of Shanxi Medical University, Taiyuan, 030032 China; 6 Department of Digestive Oncology, Tongji Hospital, Tongji Medical College, Huazhong University of Science and Technology, Wuhan, 430030 China

**Keywords:** Delphi study, Nursing management, Index system, Core competency, Billing

## Abstract

**Aim:**

This study developed a set of competency evaluation indicators for billing nurses in China.

**Background:**

In clinical practice, nurses often take up billing responsibilities that are accompanied by certain risks. However, the competency evaluation index system for billing nurses has not been established in China.

**Methods:**

This study consisted of two main phases of research design: the first phase included a literature review and semi-structured interviews. Individual semi-structured interviews were conducted with 12 nurses in billing departments and 15 nurse managers in related departments. Concepts distilled from the literature review were linked to the results of the semi-structured interviews; this phase produced the first draft of indicators for assessing the professional competence of nurses in billing departments. In the second phase, two rounds of correspondence were conducted with 20 Chinese nursing experts using the Delphi method to test and evaluate the content of the index. The consensus was defined in advance as a mean score of 4.0 or above, with at least 75% agreement among participants. In this way, the final indicator framework was determined.

**Results:**

Using the iceberg model as a theoretical foundation, the literature review identified four main dimensions and associated themes. The semi-structured interviews confirmed all of the themes from the literature review while generating new themes, both of which were incorporated into the first draft of the index. Then two rounds of the Delphi survey were conducted. The positive coefficients of experts in the two rounds were 100% and 95%, respectively, while the authority coefficients were 0.963 and 0.961, respectively. The coefficients of variation were 0.00–0.33 and 0.05–0.24, respectively. The competency evaluation index system for billing nurses consisted of 4 first-level indicators, 16 s-level indicators, and 53 third-level indicators.

**Conclusion:**

The competency evaluation index system for billing nurses, which was developed on the basis of the iceberg model, was scientific and applicable.

**Implications for nursing management:**

The competency assessment index system for billing nurses may provide an effective practical framework for nursing administration to evaluate, train, and assess the competency of billing nurses.

## Background

Each clinical department has positions for billing, including department billing, cost inquiry, audit, and discharge settlement. However, in real practice, such responsibilities are mostly performed by department nurses, who struggle with the dual identities of being a “nurse” and “department billing clerk.” Therefore, they end up shouldering more responsibilities than expected. Medical cost is the most sensitive issue that patients care about apart from their illness. Therefore, accurate and reasonable charges ensure patients’ interests and maintain hospitals’ reputations [1]. Training qualified billing nurses can enhance their billing expertise, reduce billing errors, protect patients from receiving the maximum health insurance reimbursement, and foster trust between nurses and patients [2–3]. Studies have shown that hospitals at home and abroad do not handle medical orders, billing, and refund problems perfectly, resulting in under-billing and erroneous billing problems [4–6]. Because of developments in information technology, several hospitals have implemented electronic medical order billing and network billing [7, 8]. However, due to irregular doctor orders, inconsistent expenses and actual time, and poor understanding of pricing policies, billing remains a problem of concern [9]. Patients doubt their bills, and medical costs are also increasing rapidly [5, 10]. In recent years, the National Healthcare Security Administration has increased unannounced inspections, thereby increasing pressure on nurses. Therefore, as key team members, billing nurses need to improve their professional service capabilities [11] to promote satisfactory clinical financial practices. The *Outline of China Nursing Career Development Plan 2016–2020* and *2021–2025* proposed to establish a “demand-oriented, position competency-focused” nurse training system. Position competency is the sum of knowledge, skills, abilities, and traits that enable employees to be competent for the job position; possessing these characteristics results in excellent job performance during the implementation of the position [12, 13]. Previous studies have focused on improving the efficiency of billing systems and reducing the rates of errors and violations [14, 15]. Several hospitals have achieved this goal by implementing intelligent electronic medical orders [16]. The National Organization of Nurse Practitioner Faculties also supports the inclusion of relevant medical competencies, such as billing practices, in nurse practitioner education [17]. However, there is little training in medical billing for billing medical staff [18] and no billing research on the job competency of nurses in this post. This study’s objective is to develop a competency index system for billing nurse job based on the “iceberg model” [19] to provide clinical reference and a practical framework for nursing managers to evaluate, train, and assess the competency of billing nurses.

## Methods

### Design

The Delphi method is commonly used to collect expert opinions about real-world problems. The Delphi technique can be used to deal with replication problems in which the opinions or judgments of individual experts are treated as a whole [20]. The Delphi process consists of multiple rounds of questionnaires sent to a panel of experts [21]. This study employed a modified Delphi process to solicit expert opinion on billing post-competency indicators for nurses. The whole study consisted of two phases: (1) preresearch: the preliminary draft of the billing post competency evaluation indicators for billing nurses was designed through literature review and semi-structured interviews, which replaced the first round of traditional survey mentioned in the iceberg model. (2) Delphi stage: A Delphi questionnaire was designed on the basis of the preliminary draft, and two rounds of Delphi surveys were conducted to reach a consensus.

### Development of the first draft of the billing post-competency evaluation indicators for nurses

The databases PubMed, Medline, Web of Science, the Cochrane Library, CNKI, and Wangfang database were searched from the database’s inception to January 2022 to understand the current status of domestic and international research on nurses in billing positions and to refine the components of competency for nurses in billing positions. The methodology consisted of searching databases for relevant systematic evaluations, meta-analyses, and original papers, analyzing the titles, abstracts, keywords, and references of the papers, determining the keywords for literature search, and continuously expanding the search terms based on the retrieved articles. The main retrieval terms included “billing nurse,” “billing,” “inpatient billing,” “charge management,” “reimbursement,” “costs,” “coding,” “medicare,” “insurance,” “major medical,” “post competency,” “iceberg model,” “Delphi method,” and “index system”. We included studies on core competencies required for the billing post published in English or Chinese, and fewer relevant foreign articles fit with this study. Using the iceberg model as the theoretical basis, the following four main dimensions and corresponding themes were extracted from the initial refinement of competency words for nursing positions in billing posts: theoretical knowledge (billing regulations, billing codes, health insurance knowledge, clinical knowledge, types of medical order), basic skills (ability to operate the billing system), abilities (communication skills, critical thinking), and personal traits (professional ethics, responsibility).

Through purposive sampling and snowball sampling, two members of the subject group (JL and FH) conducted one-to-one semi-structured interviews with 12 nurses and 15 nurse leaders for about 30 min. Both interviewers underwent training related to semi-structured interviews, had a thorough understanding of the survey content, survey instruments, and interview techniques, clarified data collection and organization methods, and conducted simulations before the formal interviews. The inclusion criteria for nurses were: ① ≥5 years of clinical nursing work and at least one year or more of clinical experience in hospital billing posts; ② they would still be working as billing post nurses; and ③ voluntary participation. The inclusion criteria for the nurse leaders were: ① engaged in management for ≥ 5 years; ② currently managing a unit with billing post nurses; and ③ voluntary participation in the interview. The interviews aimed to obtain the perceptions and experiences of nurses and nursing managers’ perspectives on the competency requirements of billing-post nurses. The interview questions were developed separately and finalized after repeated discussions with team members. The interview questions for billing nurses were ① please briefly introduce your major work responsibilities; ② which of your qualities or abilities have assisted in the billing work? Please give an example; ③ what qualities or abilities do you think should be possessed by an excellent billing nurse? Can you tell me a little bit about each of these areas in terms of knowledge, skills, abilities, and personal traits? Is there anything else you think you would like to share with me on the topic of billing-post nurses? Interview questions for head nurses were ① what are the qualities or abilities of an excellent billing nurse you have observed at work? Please give an example; ② what qualities or abilities do you think are required to become an excellent billing nurse? Can you tell me a little bit about each of these areas in terms of knowledge, skills, abilities, and personal traits? Is there anything else you think you would like to share with me on the topic of billing-post nurses? The sample size of the interview questions was based on the repeated data of the interviewees, and no new topics were discussed. Through semi-structured interviews, we identified all themes from the literature review phase. We extracted new themes about the iceberg model framework: familiarity with the types and prices of commonly used consumables, cost query verification, appropriate cost reminder, ability to coordinate planning, emergency response to billing errors, affinity, meticulous consistency, and self-confidence. Four primary indicators, fifteen secondary indicators, and forty-three tertiary indicators comprised the first draft of the competency indicators for nurses in billing positions, which was compiled using the information obtained from the interviews and the literature review. Four experienced nursing experts with billing post-tested the readability and feasibility of the preliminary draft.

### Delphi process

#### The expert panel

The expert panels participating in the Delphi survey were from different regions and organizations in China. Inclusion criteria included ① education background: bachelor’s degree and above; ② professional title: intermediate and above; ③ work experience and years: engaged in clinical nursing, nursing management, or financial work in hospitals for 10 years or more in a department with billing post or work experience as a billing nurse; ④ familiarity with the Delphi method; and ⑤ voluntary participation. A total of 20 experts were ultimately included. Among them, 15 were engaged in nursing management, accounting for 75% of experts, 4 were engaged in clinical nursing, accounting for 20% of experts, and 1 was engaged in hospital financial work, accounting for 0.05% of experts. The average age was 44.8 ± 4.2 years, and the average work experience was 24.1 ± 4.9 years. The Delphi expert panel demographics are listed in Table 1.

**Table 1:** Demographic data of expert panel

Characteristics	Round 1(n = 20) n (%)	Round 2(n = 19) n (%)
Gender		
Male	1(5.00)	1(5.26)
Female	19(95.00)	18(94.74)
Age(years)		
< 40	3(15.00)	3(15.79)
40–50	15(75.00)	15(78.95)
> 50	2(10.00)	1(5.26)
Educational background		
Bachelor’s degree	15(75.00)	14(73.68)
Master’s degree	5(25.00)	5(26.32)
Profession titles		
Senior	1(5.00)	1(5.26)
Associate senior	18(90.00)	17(89.47)
Intermediate	1(5.00)	1(5.26)
Professional experience(years)		
10-<20	5(25.00)	5(26.32)
20–30	13(65.00)	13(68.42)
> 30	2(10.00)	1(5.26)
Mentor type		
Master supervisor	14(70.00)	13(68.42)
others	6(30.00)	6(31.58)

#### Data collection

Based on the preliminary draft, a questionnaire on billing post competencies of nurses for experts was designed; the questionnaire contained three sections, namely the general information of experts, the main body, and a self-evaluation form for experts. In the questionnaire, the importance of each indicator was evaluated using a 5-point Likert Scale, from a 5 (very important) to 1 (very unimportant) rating scale; a column of “revision opinions” and “additional indicator suggestions” were included. We used anonymous comments, i.e., experts do not meet, experts do not know each other. They can only communicate with the investigator, and each expert can independently propose their additions, deletions, and modifications or suggest additional indicators not considered in the questionnaire. In May-June 2022, two rounds of Delphi expert correspondence were conducted, and both rounds were distributed via email by the subject leader with the experts’ informed consent. After distribution, we had full communication with the experts, explaining the purpose and requirements of our correspondence, clarifying any questionable entries, and requesting that they return the questionnaire via email within one week. The weight of the competency indicator index [22] was calculated using the Delphi method, and the indicator with a mean value ≥ 4 was considered important. The coefficient of variation ≤ 25% was considered as the screening criteria, and at least 75% consistency was noted among experts. To prevent important indicators from being deleted, only one indicator meeting the deletion criteria was to be removed after discussion with the research team. We considered expert opinions and added, merged, and revised some items; subsequently, the billing-post competency evaluation index for nurses was developed.

### Data analysis

Statistical analysis was performed using the SPSS 25.0 software. Descriptive analysis was performed using mean values, standard deviation, coefficient of variation, and proportion. The degree of experts’ activeness was expressed using the effective return rate of the questionnaire. Expert authority was assessed on the basis of judgment and experts’ familiarity with the questions, and Kendall’s coefficient of concordance was used to represent the degree of expert opinion coordination. The present study included indicators with an average score of 4 or above obtained from at least 75% of the experts.

#### Ethical considerations

This study was approved by the medical ethics committee of this hospital (Ethics No. YXLL-2022–059). The participants provided their informed consent; moreover, they were assured of their data’s anonymity and confidentiality.

## Results

### Reliability of expert questionnaire results

#### Degree of activeness of experts

In this study, 20 questionnaires were distributed to the expert panels in each round, and 20 and 19 valid responses, respectively, were obtained. The effective return rates for each round were 100% and 95%, respectively, and the total return rate was 97.5%. In the first round of the survey, 17 experts (accounting for 85% of total experts) provided their specific opinions, while in the second round, 12 experts (accounting for 63.16% of total experts) put forward their opinions.

#### Authority coefficient of experts

The authority coefficient of experts (Cr) was calculated on the basis of their judgment-making ability (Ca) and familiarity with the surveyed indicators (Cs); Cr was calculated by using the following formula: Cr =(Ca + Cs)/2. In this study, the Cas of the expert panels in both rounds of the survey were 0.985 and 0.984, respectively, while the Css were 0.940 and 0.937, respectively. The authority coefficient Cr was 0.963 and 0.961, respectively.

#### Coordination degree of expert opinions

The coordination degree of expert opinions was expressed by the coefficient of variation (CV) and coordination coefficient. The CVs of the two rounds of the survey were 0.00–0.33 and 0.05–0.24, respectively. The coordination coefficient was evaluated using Kendall’s coefficient of concordance. The coordination coefficients of the indicators in both rounds of the survey were 0.305–0.370 and 0.229–0.306, respectively. Based on Kendall’s coefficient of concordance, we calculated the p-value of the first, second, and third-level indicators as 0.000, which was statistically significant. These results revealed the optimal coordination degree of the expert opinions (Table 2**)**.

**Table 2:** Expert coordination coefficients

Items	Indicators	Kendall’s coefficient of concordance	Chi-square	*P* values
First round				
First level indicators	4	0.370	22.200	0.000
Second level indicators	15	0.305	85.397	0.000
Third level indicators	43	0.314	263.977	0.000
Second round				
First level indicators	4	0.306	17.423	0.000
Second level indicators	16	0.272	77.391	0.000
Third level indicators	53	0.229	225.890	0.000

### Results of the Delphi survey

#### Results of the first round of the Delphi survey

In the first round of the Delphi survey, experts rated the preliminary draft of the billing-post competency indicators for nurses (4 first-level indicators, 15 s-level indicators, and 43 third-level indicators; Table 3**)**. The first round of the survey received 57 comments on adding or removing competency indicators, splitting some indicators, merging duplicate indicators, and adjusting the wording at the first level. The research team carefully reviewed and discussed the expert advice, and the indicators were revised and refined accordingly. Two additional indicators were rejected because they were beyond the research scope of this study. Fourteen indicators were modified or divided. The tertiary indicator, “medical insurance knowledge”, was divided into secondary indicators. Five tertiary indicators of medical insurance type, fee standard, reimbursement policy, payment method, and fee control policy were added. Under “consumable-related information”, three tertiary indicators were added, namely, the application of consumables in the department, the content of consumables required for common operations in the department, and the common high-value consumables in the relevant departments. In “expense query and auditing ability”, “main expense checking of relevant departments” was added. The new indicators of “communication ability” included “advance communication of expenses on medical care” and “payment collection cooperating with clinicians”. In addition, the wording of one level 3 indicator was revised for “communication ability” and one level 3 indicator for “affinity”. The item “expense auditing as per medical orders”, which did not reach the 75% agreement level, was deleted after discussion because of its inclusion with “daily and discharge audit of inpatient expenses”. All reserved indicators were included in the second round of the expert survey.

**Table 3:** Results of the first round of the Delphi survey

Subjects	Mean ± SD	CV	Proportion scored ≥ 4(%)	weight
1 Theoretical knowledge	4.80 ±0.62	0.13	90.00	0.2697
1.1 Basic accounting knowledge	4.60 ±0.82	0.18	80.00	0.0672
1.1.1 Department charging criteria for common diseases/operations	5.00 ±0.00	0.00	100.00	0.0248
1.1.2 Common billing codes	4.35 ±0.88	0.20	75.00	0.0216
1.1.3 Common expense connotation	4.60 ±0.68	0.15	90.00	0.0229
1.1.4 Medical insurance knowledge	4.55 ±0.76	0.17	85.00	0.0226
1.1.5 Reimbursement requirements and procedures	4.90 ±0.45	0.09	95.00	0.0243
1.2 Characteristics of department medical orders	4.25 ±0.85	0.20	75.00	0.0621
1.2.1 Common department medical orders	4.80 ±0.62	0.13	90.00	0.0238
1.2.2 Specific billing items corresponding to each medical order	4.60 ±0.82	0.18	80.00	0.0229
1.3 Consumable-related information	4.15 ±0.49	0.12	95.00	0.0606
1.3.1 Common consumable types	4.70 ±0.73	0.16	85.00	0.0233
1.3.2 Common consumable models	4.75 ±0.64	0.13	90.00	0.0236
1.3.3 Common consumable prices	4.95 ±0.22	0.05	100.00	0.0246
1.3.4 Disposable consumables included in the main charging items or not	4.65 ±0.75	0.16	85.00	0.0231
2 Operation skills	4.65 ±0.75	0.16	85.00	0.2612
2.1 Billing skills	5.00 ±0.00	0.00	100.00	0.0730
2.1.1 Correct billing by codes	5.00 ±0.00	0.00	100.00	0.0248
2.1.2 Setting billing templates	5.00 ±0.00	0.00	100.00	0.0248
2.1.3 Appropriate refund	4.60 ±0.82	0.18	80.00	0.0229
2.2 Expense query and audit ability	4.95 ±0.22	0.05	100.00	0.0723
2.2.1 Inpatient expense query	4.45 ±0.89	0.20	75.00	0.0221
2.2.2 Use of cost query APP for patients	4.80 ±0.62	0.13	90.00	0.0238
2.2.3 Daily and discharge audit of inpatient expenses	4.50 ±0.89	0.20	75.00	0.0224
2.2.4 Expense auditing as per medical orders	3.80 ±1.24	0.33	60.00	0.0189
2.2.5 Double check expenses with the patient and family members	4.70 ±0.73	0.16	85.00	0.0233
2.3 Ability to remind payment settlement	4.40 ±0.75	0.17	85.00	0.0643
2.3.1 Appropriate ways of payment settlement	5.00 ±0.00	0.00	100.00	0.0248
2.3.2 Response to unsettled payment	5.00 ±0.00	0.00	100.00	0.0248
2.4 Device operation capability	4.90 ±0.31	0.06	100.00	0.0716
2.4.1 Billing system operation capability	4.80 ±0.62	0.13	90.00	0.0238
2.4.2 Printer operation capability	4.85 ±0.49	0.10	95.00	0.0241
3 Comprehensive ability	4.05 ±0.60	0.15	85.00	0.2275
3.1 Critical thinking	4.80 ±0.52	0.11	95.00	0.0701
3.1.1 Consistency auditing of billing and medical orders	4.35 ±0.88	0.20	85.00	0.0216
3.1.2 Identification of incorrect medical orders	4.60 ±0.75	0.16	85.00	0.0229
3.1.3 Distinguish medical insurance fraud	4.70 ±0.73	0.16	85.00	0.0233
3.2 Communication ability	4.45 ±0.83	0.19	80.00	0.0650
3.2.1 Ability to communicate related costs with patients	4.90 ±0.45	0.09	95.00	0.0243
3.2.2 Ability to interpret doubt costs with patients	5.00 ±0.000	0.00	100.00	0.0248
3.3 Overall planning ability	4.90 ±0.31	0.06	100.00	0.0716
3.3.1 Preferred billing for discharge patients	4.70 ±0.66	0.14	90.00	0.0233
3.3.2 Preferred billing for special cases	4.70 ±0.73	0.16	85.00	0.0233
3.4 Emergency response ability	4.65 ±0.67	0.14	90.00	0.0679
3.4.1 Emergency handling ability to under billing	4.55 ±0.76	0.17	85.00	0.0226
3.4.2 Emergency handling ability to erroneous billing	4.60 ±0.82	0.18	80.00	0.0229
3.4.3 Emergency handling ability to over billing	5.00 ±0.00	0.00	100.00	0.0248
4 Personal traits	4.30 ±0.57	0.13	95.00	0.2416
4.1 Value of concept	4.50 ±0.83	0.18	80.00	0.0657
4.1.1 Honesty and integrity	4.00 ±0.00	0.00	100.00	0.0199
4.1.2 Clean and self-discipline	4.50 ±0.69	0.15	90.00	0.0224
4.1.3 Meticulousness	5.00 ±0.00	0.00	100.00	0.0248
4.2 Affinity	4.05 ±0.51	0.13	90.00	0.0592
4.2.1 Pronunciation, speed, and intonation during communication	5.00 ±0.00	0.00	100.00	0.0248
4.2.2 Gentle and friendliness	4.80 ±0.62	0.13	90.00	0.0238
4.2.3 Team spirit	4.70 ±0.73	0.16	85.00	0.0233
4.3 Careful and prudent	4.65 ±0.75	0.16	85.00	0.0679
4.3.1 Patient and meticulous	4.30 ±0.66	0.15	90.00	0.0214
4.3.2 Calmness	4.05 ±0.51	0.13	90.00	0.0201
4.4 Self-confidence	4.20 ±0.62	0.15	90.00	0.0614
4.4.1 Positive to self-evaluation and work	4.70 ±0.73	0.16	85.00	0.0233
4.4.2 Confident in current post	4.80 ±0.62	0.13	90.00	0.0238

#### Results of the second round of the Delphi survey

In the second round of the survey, all indicators were finalized according to the predefined criteria, and no new items were proposed. Finally, 4 first-level indicators, 16 s-level indicators, and 53 third-level indicators were determined (Table 4**)**.

**Table 4:** Results of the second round of the Delphi survey

Subjects	Mean ± SD	CV	Proportion scored ≥ 4(%)	weight
1 Theoretical knowledge	4.37 ±0.60	0.14	94.74	0.2434
1.1 Basic accounting knowledge	4.89 ±0.46	0.09	94.74	0.06823
1.1.1 Department charging criteria for common diseases/operations	4.84 ±0.37	0.08	100.00	0.0195
1.1.2 Common billing codes	4.79 ±0.42	0.09	100.00	0.0193
1.1.3 Common expense connotation	4.42 ±0.69	0.16	89.47	0.0178
1.1.4 Reimbursement requirements and procedures	4.74 ±0.56	0.12	94.74	0.0191
1.2 Characteristics of department medical orders	4.74 ±0.56	0.12	94.74	0.06603
1.2.1 Common department medical orders	4.53 ±0.77	0.17	84.21	0.0183
1.2.2 Specific billing items corresponding to each medical order	4.74 ±0.56	0.12	94.74	0.0191
1.3 Medical insurance knowledge	4.47 ±0.51	0.11	100.00	0.0624
1.3.1 Common medical insurance types	4.84 ±0.50	0.10	94.74	0.0195
1.3.2 Common medical insurance charge criteria	4.74 ±0.65	0.14	89.47	0.0191
1.3.3 Medical insurance reimbursement policy	4.79 ±0.54	0.11	94.74	0.0193
1.3.4 Medical insurance payment ways	4.89 ±0.32	0.06	100.00	0.0198
1.3.5 Medical insurance cost control policy	4.89 ±0.32	0.06	100.00	0.0198
1.4 Consumable-related information	4.05 ±0.97	0.24	78.95	0.0565
1.4.1 Common consumable types	4.63 ±0.76	0.16	84.21	0.0187
1.4.2 Common consumable models	4.79 ±0.54	0.11	94.74	0.0193
1.4.3 Common consumable prices	4.68 ±0.67	0.14	89.47	0.0189
1.4.4 Application of department consumables	4.47 ±0.77	0.17	84.21	0.0181
1.4.5 Contents of department consumables for common operations	4.74 ±0.56	0.12	94.74	0.0191
1.4.6 Common high-value consumables in relevant departments	4.84 ±0.37	0.08	100.00	0.0195
1.4.7 Disposable consumables included in the main charging items or not	3.84 ±0.50	0.13	78.95	0.0155
2 Operation skills	4.32 ±0.75	0.17	84.21	0.2405
2.1 Billing skills	4.95 ±0.23	0.05	100.00	0.0690
2.1.1 Correct billing by codes	4.42 ±0.69	0.16	89.47	0.0178
2.1.2 Setting billing templates	4.84 ±0.37	0.08	100.00	0.0195
2.1.3 Appropriate refund	4.84 ±0.37	0.08	100.00	0.0195
2.2 Expense query and auditing ability	4.84 ±0.50	0.10	94.74	0.0645
2.2.1 Inpatient expense query	4.84 ±0.37	0.08	100.00	0.0195
2.2.2 Use of cost query APP for patients	4.79 ±0.42	0.09	100.00	0.0193
2.2.3 Daily and discharge audit of inpatient expenses	4.42 ±0.51	0.11	100.00	0.0178
2.2.4 Double check expenses with the patient and family members	4.11 ±0.32	0.08	100.00	0.0166
2.2.5 Main expense checking of relevant departments	4.74 ±0.65	0.14	89.47	0.0191
2.3 Ability to remind payment settlement	4.53 ±0.77	0.17	94.74	0.0631
2.3.1 Appropriate ways of payment settlement	4.68 ±0.58	0.12	94.74	0.0189
2.3.2 Response to unsettled payment	4.74 ±0.56	0.12	94.74	0.0191
2.4 Device operation capability	4.74 ±0.65	0.14	89.47	0.0660
2.4.1 Billing system operation capability	4.89 ±0.32	0.06	100.00	0.0198
2.4.2 Printer operation capability	4.63 ±0.68	0.15	89.47	0.0187
3 Comprehensive ability	4.32 ±0.67	0.16	89.47	0.2405
3.1 Critical thinking	4.26 ±0.81	0.19	78.95	0.0594
3.1.1 Consistency auditing of billing and medical orders	4.74 ±0.65	0.14	89.47	0.0191
3.1.2 Identification of incorrect medical orders	4.74 ±0.65	0.14	89.47	0.0191
3.1.3 Distinguish medical insurance fraud	4.11 ±0.74	0.18	78.95	0.0166
3.2 Communication ability	4.58 ±0.77	0.17	84.21	0.0638
3.2.1 Advance communication of expenses on medical care	4.68 ±0.75	0.16	84.21	0.0189
3.2.2 Payment collection cooperating with clinicians	4.63 ±0.76	0.16	84.21	0.0187
3.2.3 Ability to communicate related costs with patients	4.89 ±0.32	0.06	100.00	0.0198
3.2.4 Ability to interpret doubt costs with patients	4.89 ±0.32	0.06	100.00	0.0198
3.3 Overall planning ability	4.58 ±0.90	0.20	84.21	0.0638
3.3.1 Preferred billing for discharge patients	4.68 ±0.58	0.12	94.74	0.0189
3.3.2 Preferred billing for special cases	4.42 ±0.77	0.17	94.74	0.0178
3.4 Emergency handling ability	4.37 ±0.96	0.22	78.95	0.0609
3.4.1 Emergency handling ability to under billing	4.79 ±0.42	0.09	100.00	0.0193
3.4.2 Emergency handling ability to erroneous billing	4.79 ±0.54	0.11	94.74	0.0193
3.4.3 Emergency handling ability to over billing	4.58 ±0.69	0.15	89.47	0.0185
3.4.4 Emergency handling ability to conflicts	4.79 ±0.54	0.11	94.74	0.0193
4 Personal traits	4.95 ±0.23	0.05	100.00	0.2757
4.1 Value of concept	4.79 ±0.71	0.15	94.74	0.0668
4.1.1 Honesty and integrity	4.89 ±0.32	0.06	100.00	0.0198
4.1.2 Clean and self-discipline	4.53 ±0.84	0.19	78.95	0.0183
4.1.3 Meticulousness	4.74 ±0.65	0.14	89.47	0.0191
4.2 Affinity	4.11 ±0.46	0.11	94.74	0.0572
4.2.1 Pronunciation, speed and intonation during communication	4.74 ±0.65	0.14	89.47	0.0191
4.2.2 Gentle and friendly manner	4.68 ±0.75	0.16	84.21	0.0189
4.2.3 Team spirit	4.58 ±0.77	0.17	84.21	0.0185
4.3 Careful and prudent	3.74 ±0.87	0.23	78.95	0.0521
4.3.1 Patient and meticulous	4.68 ±0.67	0.14	89.47	0.0189
4.3.2 Calmness	4.63 ±0.76	0.16	84.21	0.0187
4.4 Self-confidence	4.11 ±0.88	0.21	89.47	0.0572
4.4.1 Positive to self-evaluation and work	4.74 ±0.56	0.12	94.74	0.0191
4.4.2 Confident in current post	4.63 ±0.76	0.16	84.21	0.0187

## Discussion

### Reliability of research results

This research project’s team leader is the director of the nursing department of a tertiary hospital. As an authoritative nursing expert, the team leader is proficient in such nursing research methods. The members of the research team have scientific research experience; of them, more than 60% of the members are postgraduates, ensuring the scientific rigor of the research process. The 20 experts hold a bachelor’s degree or higher, 65% have 20 years of work experience, and 95% have senior professional titles. The department in which the panel of experts worked had a billing post or the experts had work experience in a billing post; therefore, they have a deep understanding of the billing post competencies that a nurse should be able to provide. The Delphi method is a mature method of correspondence, and the reliability of its results is mainly judged according to three aspects, namely enthusiasm, authority, and the coordination degree of experts. The higher than 70% effective return rate of the questionnaire indicated the high enthusiasm of experts. The reliability of experts is considered to be high when the authority degree of experts is 0.70 and above. The authority coefficients of the experts were 0.963 and 0.961; both values were > 0.70, indicating that the selected experts had a high authority in this field of research and the results were trustworthy. CVs of both rounds of the Delphi survey were 0.00–0.33 and 0.05–0.24, respectively, indicating that the experts’ opinions on the research content have minor fluctuation. Furthermore, the Kendall coefficient of concordance results revealed a high consistency between expert opinions, leading to acceptable research results. Furthermore, we employed consensus-based percentages to strengthen the reliability of the findings.

### Significance of constructing competency indicators for billing nurse post

Billing quality is essential for maintaining the quality of a medical institution [23]. The quality of clinical billing work is crucial as it represents the legitimate rights and interests of the country, patients, and medical staff. Medicare billing errors, billing with billing codes that are more serious than the actual disease present or more expensive than the treatment provided can harm the state; overbilling and billing for unnecessary or unprovided services harm patients. Omissions affect hospital staff’s legitimate interests negatively, so the billing post’s work is an important part of the department’s work and good financial practices that the billing nurse can implement to reduce billing errors in clinical practice [24]. Improving the efficiency and accuracy of department billing posts may benefit healthcare organizations [25]. The clinical billing post is accompanied by great responsibility as it involves detailed chores that require good communication skills [26]. In actual practice, mostly nurses undertake the department billing post. Efficient billing nurses can greatly minimize the incidences of patient distrust and disputes caused by billing problems and improve the relationship between clinicians and patients to a large extent. Moreover, a standardized indicator system can effectively improve medical billing compliance and billing nurse efficiency and reduce waste in the healthcare system [27, 28]. Unfortunately, billing competencies are not included in the core competencies of the current nursing curricula. Identifying the core competencies allows billing nurses to improve their knowledge and skill reserves before taking the post, to be competent for the post, and improve billing compliance. It can also reduce erroneous billing and risk exposure and provide a reference for supervisors for evaluating nurses’ abilities, providing appropriate training and assessment.

### Content analysis of the developed competency indicators for billing nurse post

The current work developed a set of core competency indicators for billing nurses in China on the basis of the “iceberg model” and defined a series of knowledge, skills, and personal traits essential for billing-post competency.

#### First-level indicators

In the iceberg model, the knowledge and skills “above the iceberg” are the necessary abilities to effectively complete the work. These abilities belong to the basic competency category and are easy to improve and cultivate. Personal traits “below the iceberg” are the competency qualities that distinguish whether an individual is excellent or not, and these qualities belong to the competency identification category. The iceberg model has been widely applied and recognized in nursing as [29] it is suitable for evaluating core competencies. The four first-level indicators of theoretical knowledge, operation skills, comprehensive ability, and personal traits determined in this study are relatively scientific and rational; moreover, it conforms to the framework of the iceberg model.

#### Second-level indicators

We conducted two rounds of the Delphi survey, and a total of 16 s-level indicators were determined. Knowledge and skills mainly included basic billing skills, characteristics of doctor’s orders, consumable-related information, expense query, checking, and payment urging that includes both medical background information and some financial knowledge. These indicators represent the basic requirement for eligible billing nurses and are necessary for the effective completion of tasks; therefore, these indicators are highlighted in hospitals at present. Some studies have mentioned that billing staff must be familiar with billing codes to ensure proper billing due to unfamiliarity with billing knowledge in health systems, which can lead to unintentional abuse and exposure to serious legal sanctions [3, 30], which is consistent with the basic accounting knowledge in our metrics. In addition to basic billing, medical expertise is even more critical to healthcare organizations’ billing practices; for example, incorrect medical diagnosis coding can lead to lost revenue and negatively impact the delivery of care [31], so medical-related knowledge such as medical order characteristics is covered in our indicators. To the best of our knowledge, medical insurance regulates medical service and clinical billing behaviors, which are important aspects of practice management. Medical insurance affects the financial stability of medical institutions, promotes the improvement of medical service quality [32], and safeguards the vital interests of patients. As health insurance policies continue to innovate and develop, billing policies and reimbursement processes vary greatly from one medical institution to another. The lack of knowledge about health insurance payment and cost control may also affect the normal operation of hospitals [33–34]. Therefore, we adopted the expert opinion and moved the “medical insurance knowledge” from the third-level to the second-level indicators, adding five corresponding third-level indicators. Moreover, “emergency response ability” was changed to “emergency handling ability.” Some experts assumed that response ability is often reflected while clearing doubts about patient expenses; therefore, it should be termed “communication ability.” On the basis of the results of two rounds of the Delphi survey, personal traits were determined as four second-level indicators, namely value of concept, affinity, meticulousness, and self-confidence. The four second-level indicators were potential abilities to acquire knowledge and skills, indicating the importance of implicit features; this result is in line with the connotation of the iceberg model. Some studies have discussed medical billing in terms of ethics, mentioning that although medical ethics is an accepted part of medical education, the financial aspects of medical practice are almost never discussed. Therefore, healthcare professionals have a great deal of latitude in billing services, and such noncompliant billing practices to “maximize” or “optimize” financial returns are unethical and require education and regulation [35–36]. Such second-level indicators cover all the tasks of billing nurses, allowing nursing staff at all levels to fully understand the post quickly.

#### Third-level indicators

The supply of consumables is an important and increasing component of hospital costs [37], so we included indicators related to consumables. Regarding “consumable-related information,” some experts considered that billing nurses should get familiar with the types of consumables used in each operation to prevent underbilling. Moreover, billing nurses should pay more attention to the management of high-value consumables. Hence, we accepted the expert opinions and added the following components to the third-level indicators: “contents of department consumables for common operations,” “application of department consumables,” and “common high-value consumables in relevant departments.” Because expense auditing is crucial, “expense query and auditing ability” was added regardless of whether the hospital adopted the information system for billing or not. Experts argued that ‘expense audits according to medical orders’ are crucial; however, it was concerned with ‘daily and discharge audits of inpatient expenses’ and was removed after group discussion. Some experts further proposed that billing nurses should not only pay attention to the expenses of their own departments but also help to audit the main expenses of related departments, such as settlement and discharge of patients. Therefore, “main expense checking of relevant departments” was added to the third-level indicators. Some experts believe that billing nurses should not only know how to communicate with patients but also know how to communicate and cooperate with clinicians and nurses; moreover, they must be capable of certain response abilities. Therefore, the items of “advance communication of expenses on medical care” and “payment collection cooperating with clinicians” were added. The three-level indicators clearly indicate the characteristics and details of the billing post, thereby allowing nurses to clarify the tasks and ability requirements before taking up the billing position. The indicators would also help teachers conduct training classes, and managers carry out the evaluation process.

### Limitations

Due to issues such as research funding and time constraints, we could send the questionnaire to only 20 experts from different regions of China. The expert panels exhibited optimal authority, reliability, and representation; however, the opinions may not represent the overall view. In addition, the post-competency billing indicators developed in this study require further tests to validate their clinical applicability. Because the billing post and charging system of nurses varies in different hospitals, the required competencies may vary accordingly.

## Conclusion

The present study constructed scientific and credible competency evaluation indicators for billing nurses on the basis of the iceberg model. The indicators included 4 first-level indicators, 16 s-level indicators, and 53 third-level indicators. The adoption of these indicators may play an important role in the selection, training, assessment, and evaluation of nursing staff in this position.

### Implications for nursing management

Billing nurses are necessary for every clinical department, and nursing managers should develop and adopt a standard to evaluate their work and guide the clinical practice of nurses. The constructed billing-post competency indicators for nurses satisfy this requirement and provide a framework for selecting, evaluating, and training billing nurses. Moreover, it can improve the quality of nursing management and reduce the economic risk during the process of medical care. This is especially crucial because the economic risks of health care are inseparable from the clinical consequences of health care.

## Data Availability

All data generated or analyzed during this study are included in this published article.
